# Automated and controlled mechanical stimulation and functional imaging *in vivo* in *C. elegans* †
†Electronic supplementary information (ESI) available. See DOI: 10.1039/c7lc00465f



**DOI:** 10.1039/c7lc00465f

**Published:** 2017-06-13

**Authors:** Yongmin Cho, Daniel A. Porto, Hyundoo Hwang, Laura J. Grundy, William R. Schafer, Hang Lu

**Affiliations:** a School of Chemical & Biomolecular Engineering , Georgia Institute of Technology , USA . Email: hang.lu@gatech.edu; b Interdisciplinary Bioengineering Program , Georgia Institute of Technology , USA; c Medical Research Council Laboratory of Molecular Biology , Cambridge , UK

## Abstract

*C. elegans* is a useful genetic model system for investigating mechanisms involved in sensory behavior which are potentially relevant to human diseases. While utilities of advanced techniques such as microfluidics have accelerated some areas of *C. elegans* sensory biology such as chemosensation, studies of mechanosensation conventionally require immobilization by glue and manual delivery of stimuli, leading to low experimental throughput and high variability. Here we present a microfluidic platform that precisely and robustly delivers a wide range of mechanical stimuli and can also be used in conjunction with functional imaging and optical interrogation techniques. The platform is fully automated, thereby greatly enhancing the throughput and robustness of experiments. We show that the behavior of the well-known gentle and harsh touch neurons and their receptive fields can be recapitulated. Using calcium dynamics as a read-out, we demonstrate its ability to perform a drug screen *in vivo*. We envision that this system will be able to greatly accelerate the discovery of genes and molecules involved in mechanosensation and multimodal sensory behavior, as well as the discovery of therapeutics for related diseases.

## Abstract 2

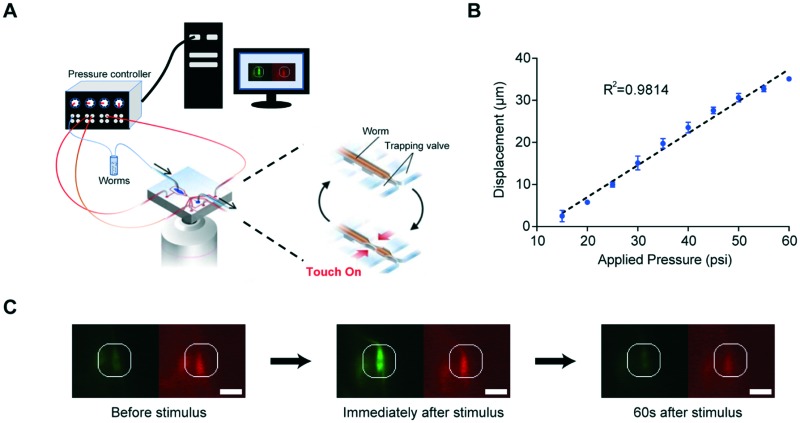
A new automated microfluidic platform can deliver a wide range of mechanical stimuli for functional neural imaging in *C. elegans*.

## Introduction

*Caenorhabditis elegans* has been an important model system for the elucidation of genetic and cellular mechanisms in sensory behavior. One example is mechanosensation, one of the earliest studied circuits in *C. elegans*, which is required for multiple sensory modalities such as touch, hearing, and balance, and is linked to a multitude of human disorders including deafness.^1–4^ Molecular mechanisms responsible for mechanotransduction have been partially elucidated using a variety of model organisms.^5,6^ Due to the ease of genetic manipulation in *C. elegans*, several genes involved in mechanotransduction have been identified.^7–14^ Furthermore, the neuronal circuitry involved in mechanosensation has been identified.^15–18^ Conventional mechanosensation experiments with *C. elegans* involve manual delivery of a mechanical stimulus to anterior or posterior regions of animals via an eyebrow hair or a metal pick^11,15,17,19^ and visual scoring of touch avoidance behavior, an assay subject to considerable variability between experimenters. Computer-controlled stimulation methods, for example using a piezo-driven micro-stylus, have been used with electrophysiological and functional imaging approaches to deliver more repeatable mechanical stimuli to animals.^9,20–24^ However, recording of neuronal responses by patch clamping or calcium imaging in response to precisely controlled mechanical stimulation requires animals to be immobilized with glue,^9,20–22,24,25^ limiting the experimental throughput and disallowing the recovery of animals for screens or further experimentation. Moreover, gluing itself is likely to affect the neuronal or circuit response, and differences in the extent of gluing introduce additional experimental variability.

Microfluidics has long been used as a “lab-on-a-chip” technology, allowing for well-controlled and high-throughput experiments with small samples.^26^ In addition to enabling precise perturbations on the micron scale, microfluidic devices can easily be designed to work together with optical microscopy, allowing for imaging of fluorescent probes such as calcium indicators.^27–29^ For *C. elegans* experimentation particularly, microfluidics has been a widely adopted technology due to the match in length scale and compatibility with fluid handling.^27^ Various devices have been developed for delivering a variety of stimuli, including chemical cues,^29–34^ temperature gradients,^35–38^ and oxygen levels,^39–42^ while simultaneously recording neuronal responses through calcium imaging. In contrast, there is currently only one microfluidic system delivering mechanical stimuli to *C. elegans.* ^43^ While useful, this system only generates a response in gentle touch neurons in the buzz mode, likely due to the small amplitude of deformation the actuators can withstand. There is still an unmet need for a robust and automatable experimental platform to deliver a wide range of mechanical stimuli (*e.g.* additional modes of stimulating gentle touch neurons and stimuli strong enough to stimulate harsh touch neurons).

To address this need, we present a microfluidic platform for delivering robust and precise mechanical stimuli to *C. elegans* by using pneumatically actuated structures. The device is fully automated, minimizing human variability and improving experimental throughput. It is fully compatible with fluorescence imaging of the calcium dynamics of neurons, which enables mechanistic interrogations as well as high-throughput genetic or drug screens. Here we demonstrate the design and utility of such a system in the context of a pilot drug screen.

## Materials and methods

### Strain

*C. elegans* was maintained under standard conditions and fed OP50 bacteria.^44^ The following strains were used in this study:

AQ3236 *ljIs142[mec-4::GCaMP6m::SL2TagRFP, unc-119] II; unc-119(ed3) III*

TV17924 *wyls5007[ser2prom3::GCaMP6, egl-17::mCherry] X*

CX10979 *kyEx2865[sra-6::GCaMP3, Pofm-1::GFP]*

GT243 *aEx2[pglr-1::GCaMP6(s), punc-122::GFP]*

RW1596 *stEx30[myo-3p::GFP + rol-6(su1006)]*

To construct AQ3236, we used a single-copy insertion vector containing a GCaMP6M transgene codon-optimized for *C. elegans*, under the control of the *mec-4* promoter (a gift from Doug Kim at HHMI Janelia Research Campus). Single-copy chromosomal integrations were obtained using the MosTic procedure.^45^ Unless otherwise specified, all worms imaged in this study are adults.

### Chip design and fabrication

The device consists of a worm inlet/outlet, an imaging channel (50–60 μm deep), and four sets of actuated PDMS membranes. Animals loosely fit in the channel and are trapped (but not held) in the imaging area by two sets of actuated membranes. The width of an actuated PDMS membrane is 150 μm, the distance between the first and second sets of membranes is 200 μm and that between the second and third sets of membranes is 250 μm.

Since the worms were not immobilized using drugs, the animals' head or tail can move in the imaging channel of the microfluidic chip. This movement sometimes blurs images. To reduce the movement of the head or tail part of the worms, a three-step vertical tapering of the imaging channel was used. The thickness of the first layer was 20 μm and the second and third layers were 15 μm for the 50 μm deep imaging channel; these layers were created with an SU-8 2015 negative photoresist (MicroChem) using standard photolithographic techniques.^46^

To create the actuated PDMS structure to touch and trap worms, a multi-layer soft lithography process^47^ was used. For the bottom flow layer of features, 23 : 1 PDMS was deposited via spin coating to create a thin layer. For the top control layer, 10 : 1 PDMS was directly poured onto a blank master, which does not have any features, to create a thick and mechanically rigid handle layer. Both layers were then placed into a 90 °C oven for 25–30 minutes until the control layer PDMS was rigid but sticky. After they were manually aligned, additional 10:1 PDMS was poured and cured for several hours to create a rigid handling layer for the device.

### Calcium imaging

All imaging experiments were performed using a Leica DMIRB inverted microscope with a 40× air objective (N.A. 0.75). Video sequences were captured using a Hamamatsu EM-CCD camera with 100 ms exposure time. Simultaneous two-color imaging was performed using a DV2 beamsplitter (Photometrics) containing a GFP/RFP filter set. Excitation light for fluorescence imaging was delivered through a projector system previously developed.^48^ In experiments for the measurement of mechanosensory neuronal responses, stimuli were delivered 10 s after recording the baseline activity of neurons. In the experiment for the measurement of interneuronal recording, stimuli were delivered 30 s after recording the baseline activity of neurons. Videos were recorded for 60–180 s following stimulus delivery.

### Data analysis

Fluorescence intensities for each frame were obtained using customized neuron-tracking MATLAB scripts (Fig. S2†). In strains where both GCaMP6 and RFP are expressed, the ratio between intensity values was computed 
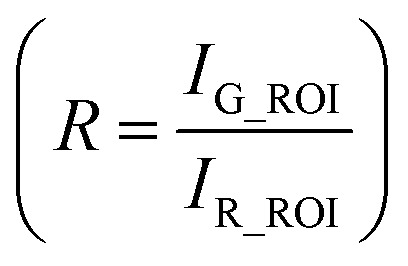
 in order to minimize movement artifacts. When only GCaMP was available, fluorescence values were computed by subtracting the background intensity (*F* = *I*_G_ROI_ – *I*_G_Back__). GCaMP and RFP intensities were measured as the mean pixel intensity of the 100 brightest pixels of a circular region of interest (ROI) of 10-pixel radius. Background intensities were subtracted to adjust for variations in lighting conditions, and were measured as the mean pixel intensity of an ROI in a background region (Fig. S2†). Calcium traces were computed as the change in *R* or *F* from the baseline value 
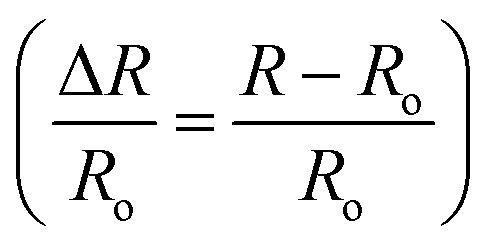
 or 
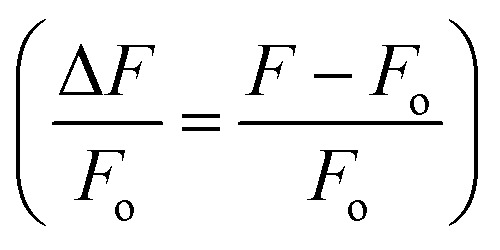
. Baseline values were computed as the mean *R* or *F* prior to stimulus delivery.

### Drug screening

Worms were roughly synchronized by picking 20–25 L4 worms and allowing them to lay eggs overnight before removing them from the plate. After two days at 20 °C, tightly age-synchronized populations of worms were obtained by washing adults and L1s off of these plates and then washing newly hatched L1s from these plates after an hour's interval. The 84 compounds of the Screen-Well Orphan library (ENZO) were used for the drug screening. 20–30 tightly-synchronized L4 worms were placed in a 48-well plate (Greiner Bio-One) with 0.5 ml OP50 bacteria (OD 5) for non-treated worms and both 0.495 ml OP50 bacteria and 0.005 ml (100 μM) drugs for drug-treated worms. After 24 hours, the worms were imaged. Among the 84 compounds in the library, we tested the effects of 13 compounds on AVM neuronal responses at three different ages (from day 1 adult to day 3 adult). These compounds were chosen arbitrarily from the orphan ligand library.

## Results and discussion

**Fig. 1:**
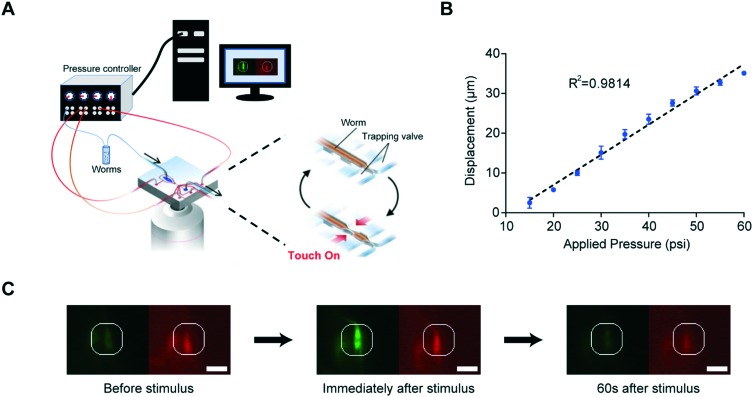
The microfluidic platform can robustly deliver a mechanical stimulus and allow imaging of calcium responses in *C. elegans* mechanoreceptor neurons. (A) An integrated system for automated functional imaging of *C. elegans* in microfluidic devices. Individual animals were sequentially loaded *via* a pressure-driven flow. The device employs multiple sets of actuated structures: valves to trap animals in a reproducible position and two sets of actuation valves used to deliver mechanical stimuli to different regions of the body. All actuators and loading procedures were automatically controlled by a customized MATLAB script. (B) Displacement of the actuated membrane by applying pressure (*n* = 4 worms). Measurements were obtained from images of transgenic worms expressing GFP along the body-wall muscle (*stEx30[myo-3p::GFP + rol-6(su1006)]*). The *R*-squared value is 0.9814. (C) Sample frames from an activated neuron show changes in fluorescence due to the mechanical stimulus. Because neurons of interest move during recordings due to the mechanical stimulus and behavioural responses, a tracking algorithm was developed to automatically record the GCaMP and RFP intensities from individual trials. Scale bar: 10 μm.

Our microfluidic device is optimized to deliver precise and repeatable mechanical stimuli to different anatomical regions of *C. elegans* (Fig. 1). Animals are loaded into an imaging channel (where the animals are not immobilized but their movement is much reduced from freely moving behavior), and mechanical stimuli are delivered through two pairs of in-plane PDMS membrane structures (Fig. 1A and S1A and B†). Two additional actuated structures act as loading and imaging valves (Fig. S1A and B†). The structures are pressure-actuated, and when deflected, exert a mechanical stimulus on animals trapped in the imaging channel (Fig. S1C†). Our design and the fabrication protocol (see Materials and methods for details) allow us to use ordinary pressure ranges (10–60 psi or 70–415 kPa) to actuate the stimulating structures without failure; furthermore, the deflection and deformation caused by these actuations are in similar ranges to conventional approaches,^20,21^ and roughly linear with the actuation pressure (Fig. 1B). This design retains animals in plane and relatively stationary but not fully immobilized, thus allowing high-quality imaging of calcium transients in cell bodies and subcellular processes (Fig. 1C). To automatically identify the fluorescently labeled neuron of interest and extract quantitative calcium transients, we developed a neuron tracking algorithm (Fig. S2†). The actuated structures are connected to a pressure source via individually controlled off-chip solenoid valves, allowing for an automated and rapid “load-and-image” routine (Fig. S1D†). Additionally, the duration and pressure of stimuli can be easily controlled, allowing for the study of a variety of behaviors upon application of mechanical stimuli such as graded response, habituation, and arousal; the anatomical location of the stimuli can be varied by the location of the stimulation structures relative to the trapping structures. Furthermore, this design can be easily adapted to allow for sorting and imaging animals of various sizes.

**Fig. 2:**
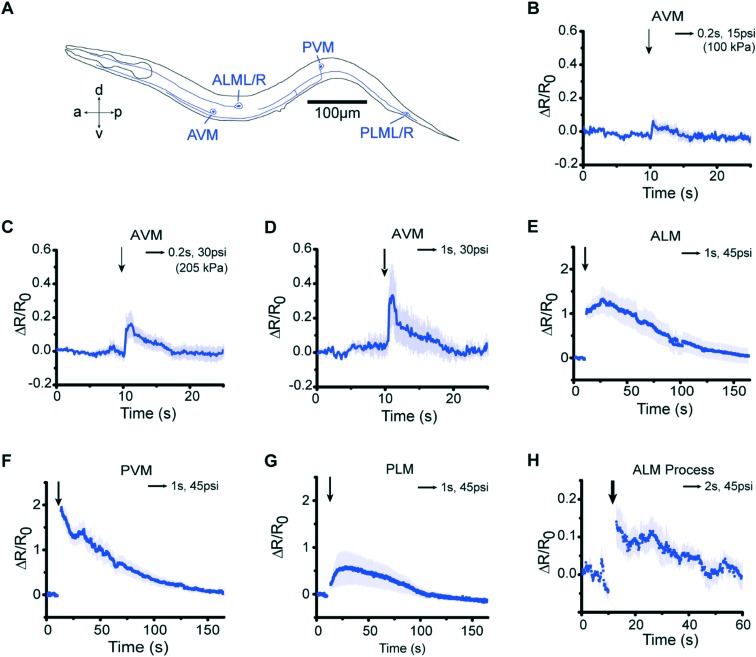
The microfluidic platform delivers mechanical stimuli emulating gentle touch. (A) Schematic of the six gentle touch neurons – AVM, ALML/R, PVM, and PLML/R. (B–D) AVM cell body responses to various stimuli with low pressures and durations: (B) 15 psi and 0.2 s (*n* = 5), (C) 30 psi and 0.2 s (*n* = 10), and (D) 30 psi and 1 s (*n* = 4). The AVM response is reduced when using lower pressures (comparing B to C). The response is also attenuated when using shorter durations (comparing C to D). Error bars represent SEM. (E–H) Responses of the *C. elegans* gentle touch neurons to mechanical stimuli. Average traces of GCaMP6 signals in (E) ALM soma to 1 s stimulus (*n* = 16), (F) PVM soma to 1 s stimulus (*n* = 17), (G) PLM soma to 1 s stimulus (*n* = 9), and (H) ALM process to 2 s stimulus (*n* = 7) at 45 psi. Error bars represent SEM. For panels (B–H), the arrow thickness indicates the stimulation duration.

To demonstrate the utility of the system, we examined the responses of the classic gentle touch receptor neurons (AVM, ALMR/L, PVM, and PLMR/L)^15,16^ (Fig. 2A). The stimulus is traditionally delivered to moving worms by an eyebrow hair or to immobilized worms by a stiff probe.^20,25^ In contrast, we deliver the mechanical stimuli to a worm that is in the imaging channel on-chip, while calcium transients in the relevant cells are measured by imaging fluorescence reported by the genetically encoded calcium indicator (GECI) GCaMP6m.^49^ We first demonstrate how the well-controlled deformation in the animal's body can translate to calcium transients. For example, by applying a short (0.2 s), gentle (15 psi) anterior stimulus, the AVM soma shows a small but detectable transient (Fig. 2B and Movie S1†), similar to previous findings in glued worms.^20^ Increasing the intensity or duration of the stimuli increases the calcium transients (Fig. 2C and D). We also observe responses on other gentle touch neurons, including ALM (Fig. 2E), PVM (Fig. 2F), and PLM (Fig. 2G and Movie S2†). Furthermore, we can also image the calcium transients in the neuronal processes, *e.g.* axon of ALM (Fig. 2H).

**Fig. 3:**
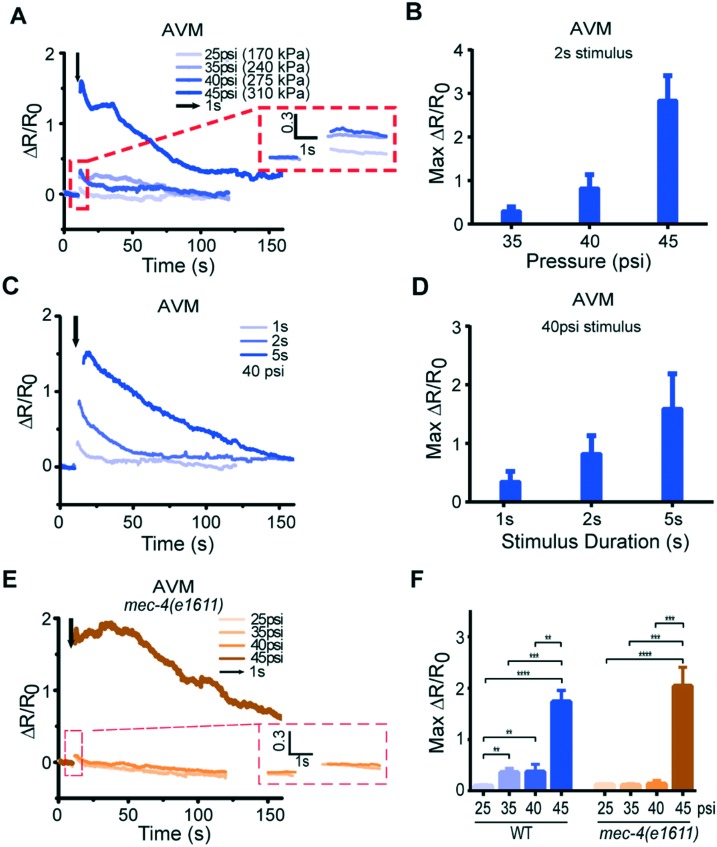
The tuneable platform enables varying levels of mechanical stimulus. (A and C) Average traces of GCaMP6 signals in AVM in response to diverse pressures and stimulus durations. (A) 1 s stimulation with diverse pressures (25 psi: *n* = 11, 35 psi: *n* = 25, 40 psi: *n* = 8, 45 psi: *n* = 27). (C) 40 psi stimulation with diverse stimulus durations (1 s: *n* = 8, 2 s: *n* = 10, 5 s: *n* = 10). (B and D) maximum responses of calcium transients correlate with (B) the applied pressure (2 s stimulus, 35 to 45 psi) and (D) the duration of stimuli (1 to 5 s stimuli, 40 psi). Error bars represent SEM. (E) Average calcium responses of *mec-4(e1611)* mutants in AVM neurons to diverse pressures with 1 s stimulus (25 psi: *n* = 18, 35 psi: *n* = 10, 40 psi: *n* = 9, 45 psi: *n* = 10). (F) Maximum responses of calcium responses of wild-type and *mec-4* mutant animals (Mann–Whitney test, *p < 0.05, **p < 0.01, ***p < 0.001, ****p < 0.0001).

Because our system delivers mechanical stimuli by applying externally controlled pressure to actuated structures, the stimuli can be regulated by the magnitude and duration of the applied pressure. We find that changing these quantitatively controlled parameters can translate to quantitative differences in calcium transients (Fig. 3A–D, Fig. S3, S4, and Movie S3†). We applied anterior stimuli of varying levels of pressure and durations, and measured calcium activity in AVM somas (Fig. 3A–D and S3†). Peak calcium transients were roughly proportional to the pressure applied (Fig. 3A and B, and S3†) and the stimulus duration (Fig. 3C and D, and S3†). We also tested AVM's responses in the well-known *mec-4*/DEG/ENaC channel mutant^9,20^ (Fig. 3E). As expected, the *mec-4* mutant gives negligible response and is insensitive to the magnitude of the stimulation input in the gentle touch regime but is responsive to harsh touch, perhaps even more so than wild-type (Fig. 3F).

**Fig. 4:**
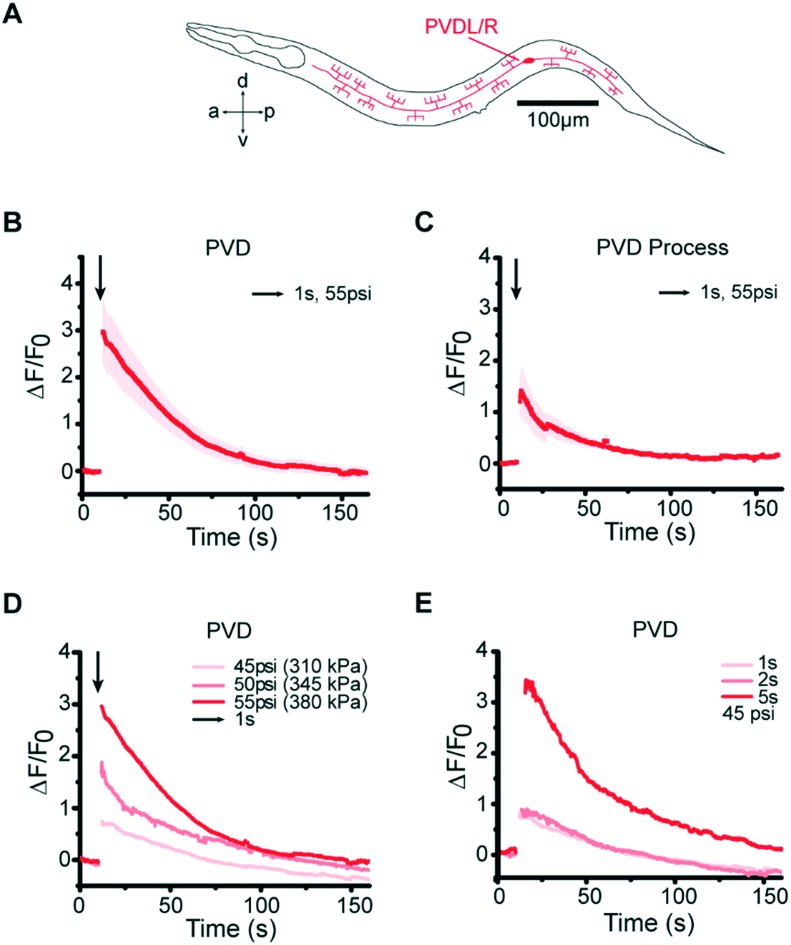
The microfluidic platform delivers mechanical stimuli emulating harsh touch. (A) Schematic of the harsh touch neurons PVDL/R. (B and C) Responses of harsh touch neurons to mechanical stimuli. Average traces of GCaMP6 signals in (B) PVD soma (*n* = 9, 55 psi) and (C) PVD process to 1 s stimulus (*n* = 5, 55 psi). Error bars represent SEM. (D and E) Average traces of GCaMP6 signals in PVD neurons in response to diverse pressures and stimulus durations. (D) 1 s stimulation with diverse pressures (45 psi: *n* = 9, 50 psi: *n* = 6, 55 psi: *n* = 9). (E) 45 psi stimulation with diverse stimulus durations (1 s: *n* = 9, 2 s: *n* = 4, 5 s: *n* = 6).

In addition to the gentle touch stimulus, our platform is also capable of delivering mechanical stimuli that activate the harsh touch neurons PVD, while using practical pressures to operate the device (Fig. 4A). This is nontrivial as any design that would have required larger operating pressure to stimulate PVD (>60 psi or 415 kPa) would have had a large failure rate that renders these experiments impractical. Our design is both robust and flexible – stimulating PVD is simply achieved by increasing the actuating pressure without changing the device design or other operating protocols. When we presented posterior stimuli of varying pressures and durations in the harsh touch regime, we observed responses in PVD neurons (Fig. 4B and C, and Movie S4†) similar to those in the gentle touch experiments: as expected, compared to gentle touch neurons, PVD required a higher pressure (55 psi) or a longer duration of stimulus (5 s) at low pressure to elicit similar responses; furthermore, PVD also shows graded response to pressure and duration (Fig. 4D and E, and S5†).

**Fig. 5:**
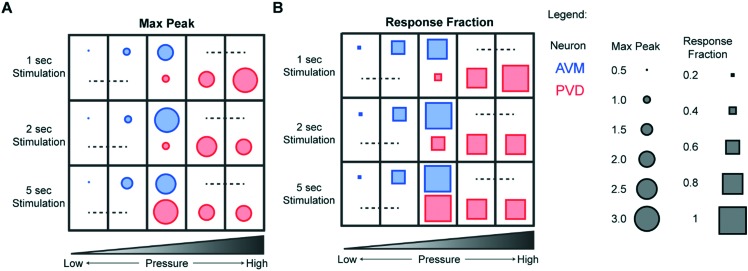
Quantitative responses of AVM and PVD under different stimulation conditions. (A and B) Peak response and response fraction of gentle touch sensing AVM and harsh touch sensing PVD in the tested parameter regimes. Each column refers to the applied pressure magnitude and each row refers to the applied duration of stimulation. For each data point, (A) the circle size indicates the max response value from 0 to 3.0 and (B) the rectangle size indicates the response fraction from 0 to 1. The response fraction is defined as the percentage of traces that shows a max response value higher than 0.5. Dashed lines indicate non-tested conditions.

Because our system can acquire data for a large number of animals more easily than conventional systems, the experiments yield information about both average responses and response rates, which is important in studying neurons under conditions with more stochastic responses. Fig. 5 summarizes a large set of experiments for both the gentle and the harsh touch neurons. Interestingly, in addition to the response magnitude (Fig. 5A), the stimulation pressure and duration also affect the response rates of both the gentle touch and the harsh touch neurons in a graded manner (Fig. 5B). For AVM, stimuli with actuation pressures higher than 40 psi produce a response rate (the fraction of animals responding) of >90%, while below 30 psi the response is more stochastic (<20%). Applying stimuli at lower actuation pressures also elicits a less sustained response or a small magnitude of response, and shorter stimuli elicits less response. PVD has a similar behavior, except that it is shifted towards the intensity of the stimuli.

**Fig. 6:**
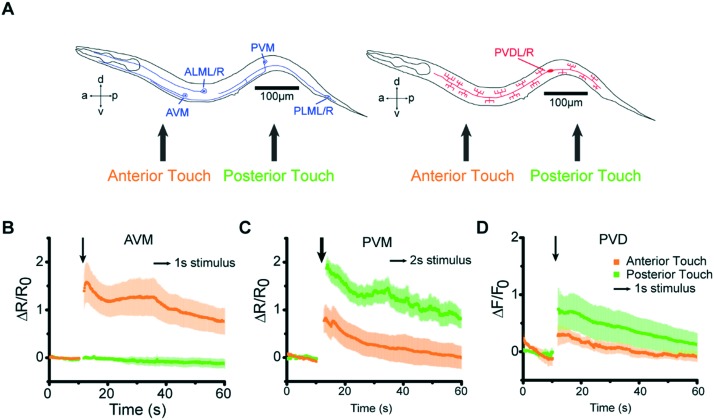
Gentle and harsh touch neurons exhibit receptive fields when a spatially resolved stimulus was delivered to the appropriate regions. (A) Schematic of the six gentle touch neurons and relative locations of mechanical stimuli. (B) The activity of AVM responds to 1 s anterior but not to posterior stimuli (anterior: *n* = 10, posterior: *n* = 5) at 45 psi. (C) Gentle touch neurons, PVM, (2 s stimulation, anterior: *n* = 10, posterior: *n* = 11) and (D) harsh touch neurons, PVD, (1 s stimulation, anterior: *n* = 9, posterior: *n* = 3) respond to both anterior and posterior stimuli at 45 psi. Error bars represent SEM. Orange denotes anterior touch and green denotes posterior touch. For panels (B–D), the arrow thickness indicates the stimulation duration. 1 s and 2 s stimulations are represented by thin and thick arrows, respectively.

Besides intensity and temporal precision, we also examined our system's ability to determine the spatial properties of mechanosensory systems (Fig. 6A). Gentle touch neurons are known to have defined receptive fields.^11,15,20,50^ Because of the small size of the stimulating structure, we can control the location of stimulation with a spatial resolution of <150 μm; the precision is controlled by the design of the devices *a priori* and the anatomy of the worm loaded for the essay, which can be controlled by age synchronization. The responses we observed were consistent with the known individual neurons' receptive fields. For example, AVM responded to anterior but not to posterior stimuli (Fig. 6B). In contrast, PVM responded to both anterior and posterior stimuli, as did PVD, with the responses to posterior stimuli being stronger for both classes of neurons (Fig. 6C and D).

**Fig. 7:**
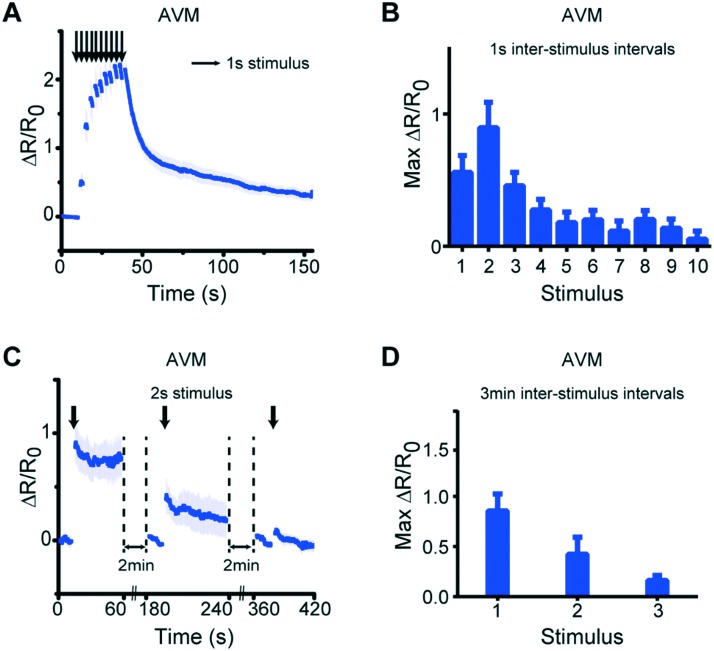
Habituation captured by delivering repeated stimuli. (A and B) When worms are exposed to 1 s stimuli with short inter-stimulus intervals (1 s), AVM neurons exhibited an incremental increase in response magnitude up to the second stimulus, and a reduced response in later stimuli (*n* = 19). (C and D) In contrast, when exposed to 2 s stimuli with long inter-stimulus intervals (3 min), the response magnitude was reduced after each stimulus (*n* = 10). Error bars represent SEM.

Besides simple stimulation, our system can also be used to deliver repeated stimuli to examine phenomena such as habituation and desensitization.^20^ To determine whether this phenomenon can be recapitulated in our system, we delivered repeated stimuli to the animals using either short (1 s) or long (3 min) inter-stimulus intervals (Fig. 7 and S6†). When receiving repeated stimuli with short intervals, the neurons exhibited an incremental increase in response magnitude up to the second stimuli, and then a reduced response in later stimuli (Fig. 7A and B, and S6†). In contrast, when using long inter-stimulus intervals, the response magnitude was reduced after each stimulus (Fig. 7C and D). These results are consistent with previous observations that habituation is dependent on the inter-stimulus duration.^20^ Thus, these experiments demonstrate how simple changes of operational parameters allow us to use the same device for a wider repertoire of the device utility.

**Fig. 8:**
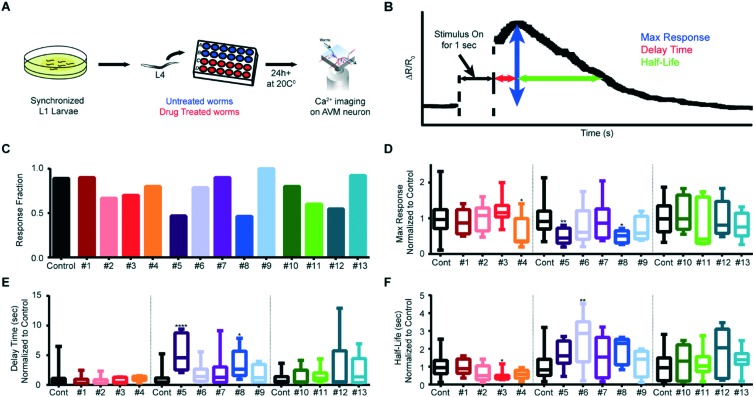
The microfluidic platform enables screens to examine compounds that may affect neuronal responses to mechanical stimuli. (A) Experimental procedure for the drug screen performed. Synchronized L1 worms are grown in NGM plates to the L4 stage and then deposited in a 48-well plate. Drug-treated worms are cultured with 0.5 ml OP50 *E. coli* bacteria (OD 5) and 100 μM drugs. Control worms are cultured with 0.5 ml OP50 *E. coli* bacteria (OD 5). Both groups of worms are incubated at 20 °C for at least 24 h. Subsequently, AVM responses to 1 s stimulus were measured on-chip. (B) Three metrics measured from individual calcium dynamic traces: maximum response, delay time (time between the end of the stimulus and the arrival of maximum response), and half-life (time it takes the response to decay to half of the maximum). (C) Fraction of animal responses upon compound treatment. (D–F) Box plots show how compounds affect specific parameters of neuronal response upon mechanical stimulation. Quantification of each response was normalized to that of the control group from the same day (day 1 adult to day 3 adult). (Kruskal–Wallis test, *p < 0.05, **p < 0.01, ***p < 0.001, ****p < 0.0001).

In contrast to gluing protocols, our system allows for automated imaging by streamlining the handling of the worms; this in turn allows for previously impractical high-throughput experiments, such as genetic or drug screens. To demonstrate the system's ability to perform rapid screens, we examined the effect of small molecules from an orphan ligand library on mechanosensation. We exposed animals in the L4 stage to the compounds, and imaged AVM activity when delivering an anterior stimulus to adult worms (Fig. 8A). Fig. 8B shows a typical response of wildtype animals without drug perturbation; calcium traces typically reach a maximum value shortly after the end of the stimulus, and then slowly decline back to baseline levels. To examine how each drug affects mechanosensation, we quantitatively compared three metrics (max Δ*R*/*R*_o_, delay time, and half-life), as well as the fraction of animals responding, between drug-treated animals and untreated animals (Fig. 8C–F and S7 and S8†). We imaged multiple rounds of adult animals exposed to 13 drugs and quantified the established parameters for the screen criteria (Table S1†). While most of the drugs screened lowered the number of animals responding to the mechanical stimulus, interestingly, a few slightly increased the response fraction (Fig. 8C). We also analyzed differences in the metrics of the calcium dynamics for drug treatment conditions and found that five of the drugs we tested affected the mechanosensation response dynamics (Fig. 8D–F, and S8†).

## Conclusions

For fundamental studies of mechanosensation, quantitative live imaging is necessary, and large sample sizes are often required to perform screens based on mechanosensory phenotypes. Our microfluidic platform allows for studying mechanosensation in *C. elegans* quantitatively and conveniently, allowing for the delivery of a variety of types of mechanical stimuli to live animals while recording neuronal activity. Experimental preparation can be accomplished for a batch of animals, so the limiting step is imaging (tens of seconds to minutes depending on the experiments); the throughput using our system can be as high as ∼10 to 100 trials per hour depending on the assay conditions. It is also straightforward to automate and run these systems in parallel to further improve the throughput. In contrast, the conventional approach (gluing worms and stimulating with a micro-stylus and a micromanipulator) generally yields ∼10 successful trials per day. The integration of hardware and software also allows for automated operations of imaging, stimulation, and quantitative analysis, further reducing potential human error and bias. This important improvement in throughput and standardization over conventional methods allowed us to conduct a pilot drug screen based on neuronal dynamics in response to mechanical stimuli, which resulted in candidates that affect the dynamics in mechanosensory neurons in a variety of ways. One can envision genetic screens performed in a similar manner to identify mechanosensory mutants. Many worm mechanosensory modalities, such as harsh touch and nose touch, involve multiple partially redundant cell types, making behavioral assays ineffective for finding genes affecting these processes. With simple integration of sorting mechanisms on chip,^51–53^ it will be possible to conduct high-throughput forward screens for mutants affecting the responses of individual neurons using a GECI-based assay. The genes identified in such screens should provide insight into the underlying mechanisms of mechanosensation, as well as find potential therapies for sensory-loss conditions such as deafness.

Because our platform employs a simple microfluidic device, it is easily adaptable for biological systems of different sizes. Scaling the devices to be smaller can allow studies of mechanosensory neurons in worm larvae during development; scaling the devices to be larger can allow studies of the mechanosensation circuit during aging in *C. elegans*, as well as neurons and circuits in other model organisms such as zebrafish or fly larvae. Lastly, because the microfluidic chip allows unhindered optical access, integrations of optogenetic methods to control cellular activity and to influence behavior^48,54–59^ can also be straightforwardly carried out in this platform, thereby greatly expanding the repertoire of biological problems to be studied.
