# Correction: automated and controlled mechanical stimulation and functional imaging *in vivo* in *C. elegans*

**DOI:** 10.1039/c7lc90104f

**Published:** 2017-10-24

**Authors:** Yongmin Cho, Daniel A. Porto, Hyundoo Hwang, Laura J. Grundy, William R. Schafer, Hang Lu

**Affiliations:** a School of Chemical & Biomolecular Engineering , Georgia Institute of Technology , USA . Email: hang.lu@gatech.edu; b Interdisciplinary Bioengineering Program , Georgia Institute of Technology , USA; c Medical Research Council Laboratory of Molecular Biology , Cambridge , UK

## Abstract

Correction for ‘Automated and controlled mechanical stimulation and functional imaging *in vivo* in *C. elegans*’ by Yongmin Cho *et al.*, *Lab Chip*, 2017, **17**, 2609–2618.



## 

The authors would like to correct errors in the genotype information of the *Caenorhabditis elegans* strain. The correct version of the strain information is shown below.

AQ3236 *ljSi2[mec-7::GCaMP6m::SL2TagRFP, unc-119] II; unc-119(ed3) III*

The Royal Society of Chemistry apologises for these errors and any consequent inconvenience to authors and readers.

