# Supplementary material for: Automated and controlled mechanical stimulation and functional imaging in vivo in C. elegans
Source: Lab Chip. 2017 Jun 13;17(15):2609–18. doi: 10.1039/c7lc00465f (PMC5575793; doi:10.1039/c7lc00465f)
Supplement: Supplementary file 1 [file LC-017-C7LC00465F-s001.pdf]

Electronic Supplementary Material (ESI) for Lab on a Chip.
This journal is © The Royal Society of Chemistry 2017

Supplemental Figures

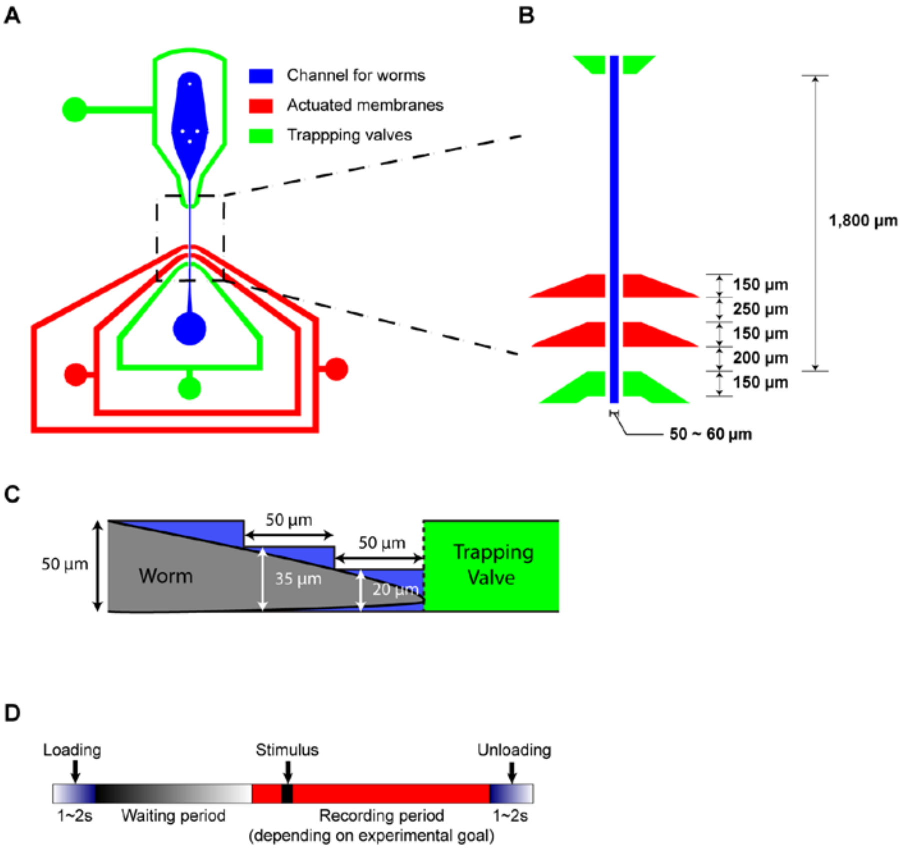

Fig. S1. Overview of microfluidic device design and dimensions.

(A and B) The device is composed of the channel for worms (50-60 µm deep and wide, which allow the animals to fit loosely inside), two sets of actuated membrane, and two sets of trapping valve. The width of both actuated PDMS membrane and trapping valve is 150 µm, the distance between first and second sets of membrane is 200 µm and second and third sets of membrane is 250 µm. (C) Diagram of scheme for restriction of the z-direction range of motion via a three-step vertical imaging channel. (D) Timeline of on-chip mechano-stimulation and functional imaging of neurons. The loading and unloading of each worm requires only a few seconds. Each animal is given a waiting period to acclimate to the environment before being stimulated and imaged. Each trial is performed by recording video to track neuronal dynamic responses and applying mechanical stimuli.

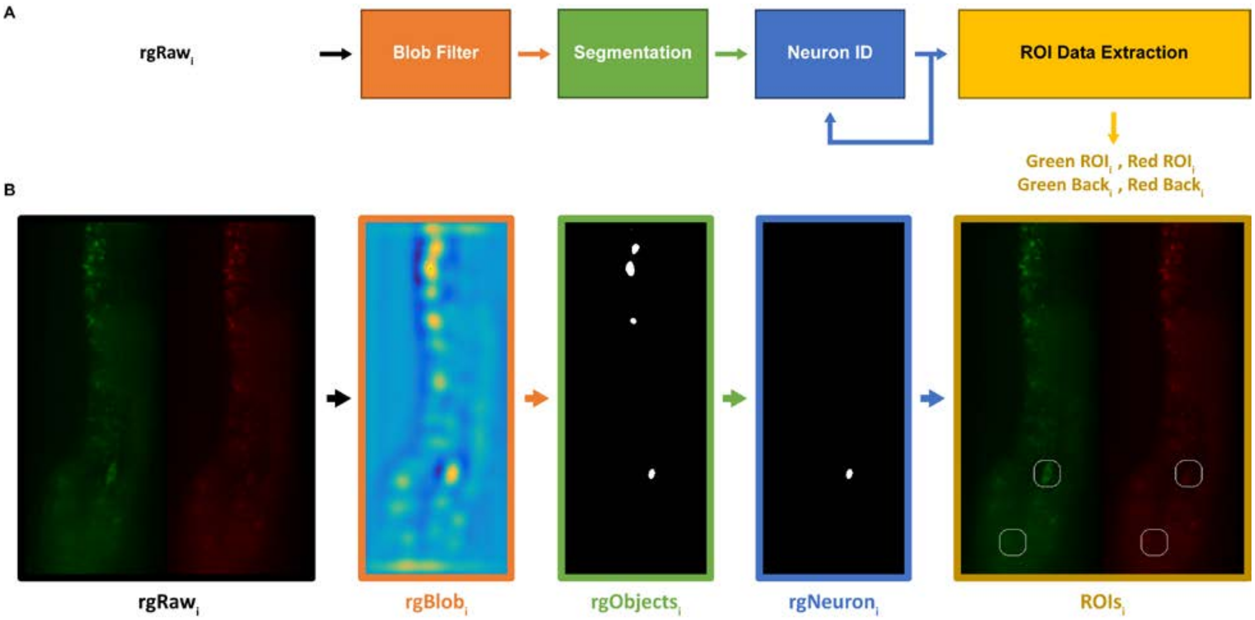

Fig. S2. Neuron Tracking Algorithm. In order to extract fluorescence intensities throughout recordings, a neuron tracking algorithm was developed. This was necessary because worms are not fully immobilized in the device, and mechanical stimuli often caused the neuron of interest to move within the field-of-view. (A) Overall schematic of the neuron tracking algorithm. For each frame *i*, raw images are processed through a blob filter (Laplace of Gaussian filter) to improve contrast and facilitate segmentation. Blob filtered images are segmented by applying an empirically determined threshold. The neuron of interest is identified by the user in the first frame, and by distance to the neuron in the previous frame. Lastly, once the neuron is detected for each frame, intensity values are extracted (Green ROI, Red ROI, Green Background, and Red Background). (B) Example of algorithm procedure.

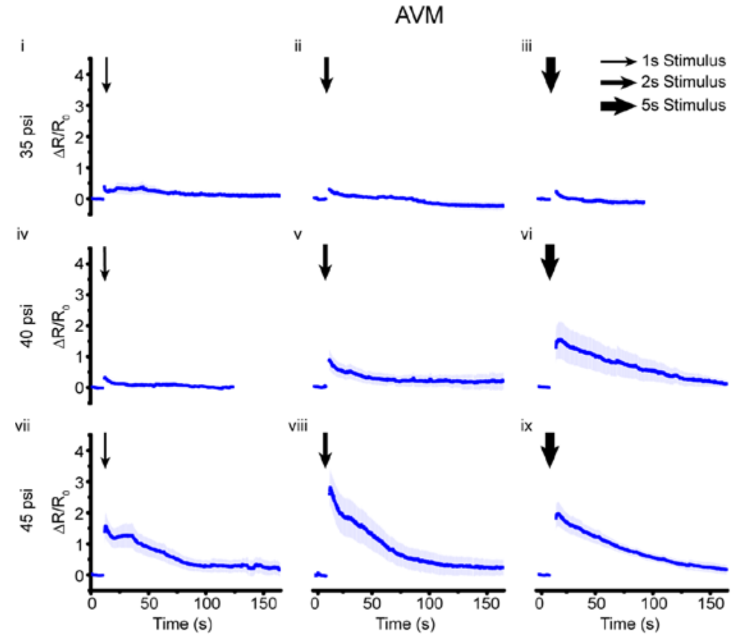

Fig. S3. Average traces of GCaMP6 signal in AVM neuron in response to diverse pressures and stimulus durations (**i-iii**: 35 psi, **iv-vi**: 40 psi, **vii-ix**: 45 psi / **i**, **iv, vii**: 1s stimulus, **ii, v, viii**: 2s stimulus, **iii, vi, ix**: 5s stimulus, sample size **i**: n=25, **ii**: n=10, **iii**: n=8, **iv**: n=8, **v**: n=10, **vi**: n=10, **vii**: n=27, **viii**: n=6, **ix**: n=10). Error bars represent SEM.

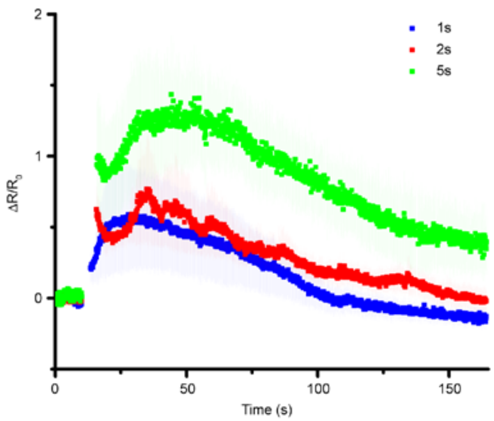

Fig. S4. PLM cell body responses to various stimulus durations (1s: n=9, 2s: n=4, 5s: n=4) at 45 psi. Similar to those of AVM, maximum responses in PLM were proportional to the stimulus duration.

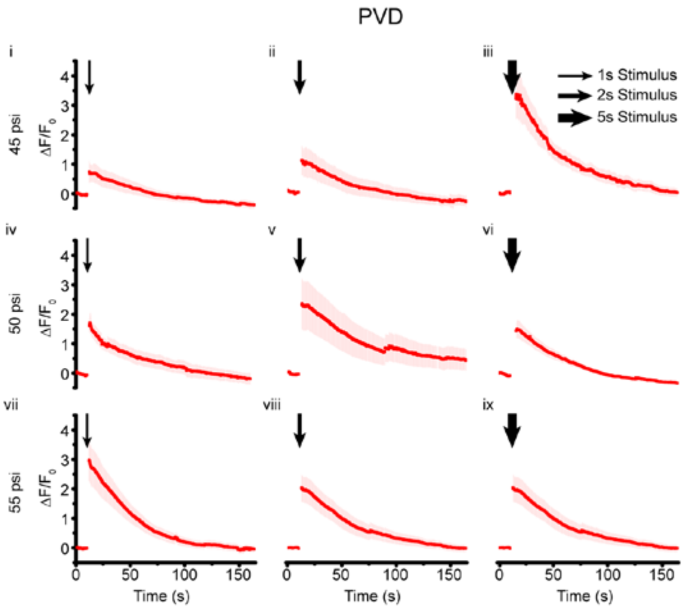

Fig. S5. Average traces of GCaMP6 signal in PVD neuron in response to diverse pressures and stimulus durations (**i-iii**: 45 psi, **iv-vi**: 50 psi, **vii-ix**: 55 psi / **i**, **iv, vii**: 1s stimulus, **ii, v, viii**: 2s stimulus, **iii, vi, ix**: 5s stimulus, sample size **i**: n=9, **ii**: n=4, **iii**: n=6, **iv**: n=6, **v**: n=9, **vi**: n=10, **vii**: n=9, **viii**: n=10, **ix**: n=10). Error bar represent SEM.

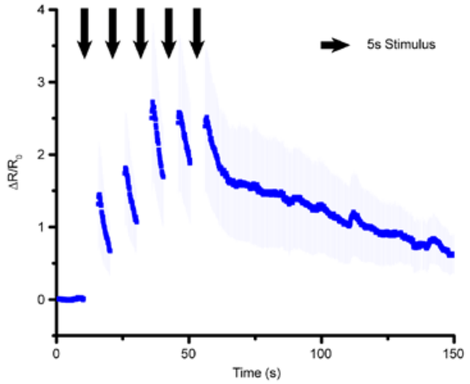

Fig. S6. AVM cell body response to delivery of repeated 5s stimuli with 5s inter-stimulus intervals (n=5). Similar to Figure 2l, traces showed incremental increases in the first few stimuli, and showed a decreased response in later stimuli.

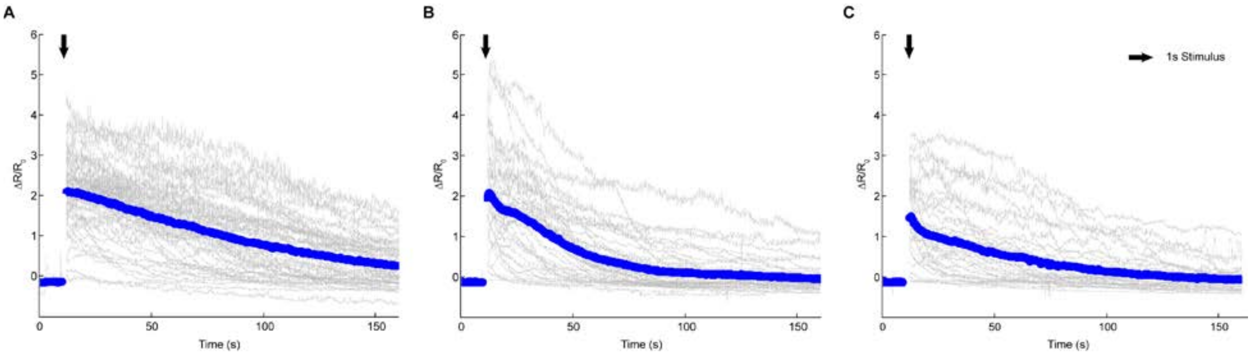

Fig. S7. Individual (gray) and average traces (blue) for AVM response in untreated animals for different control groups for drug screen. (A) Day 1 (n=53), (B) Day 2 (n=53), and (C) Day 3 (n=35) adult worms.

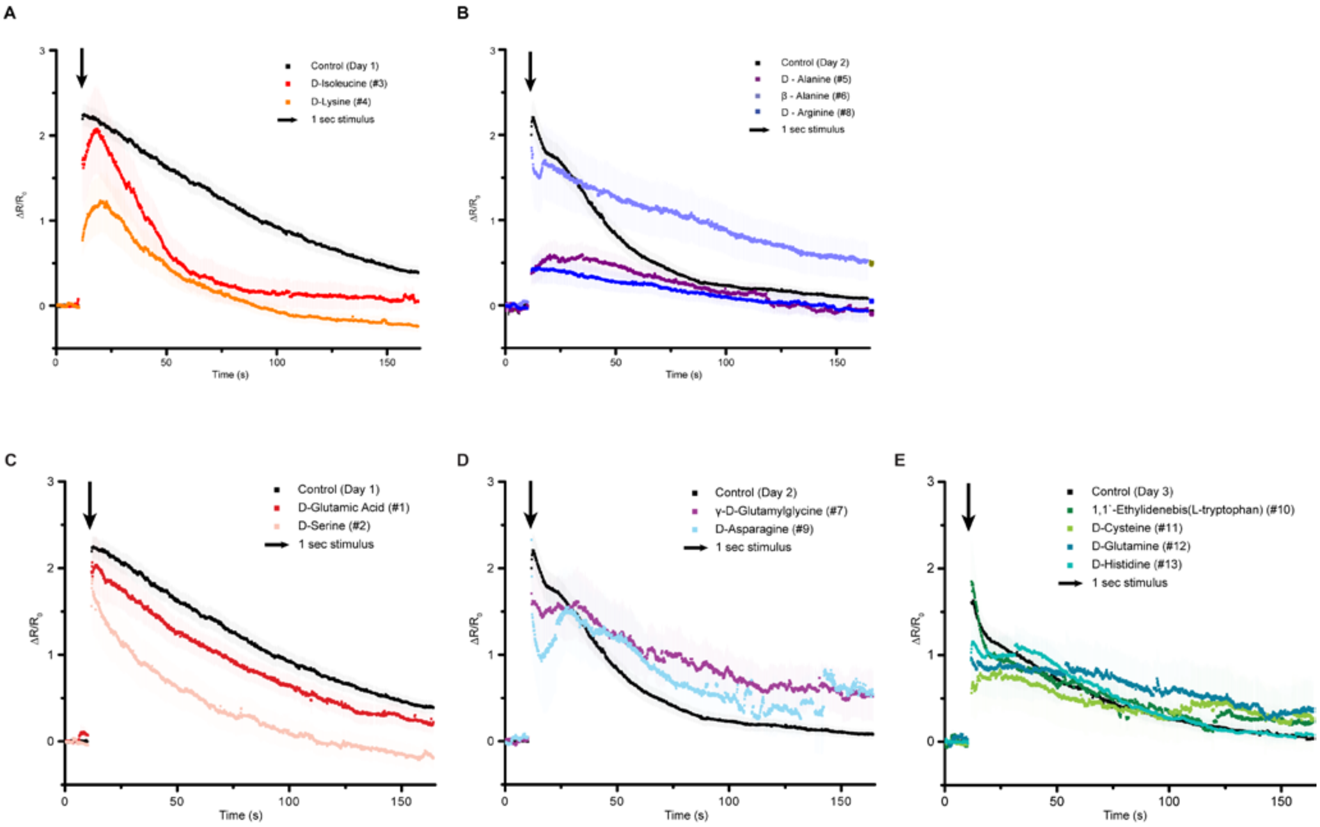

Fig. S8. Average traces for AVM response in drug-treated animals. (A and B) Average traces for AVM response in drug-treated animals that show a significant difference from the control groups. (A) Day 1 adult worms (Control Day 1: n=53, D-Isoleucine: n=10, D-Lysine: n=10). (B) Day 2 adult worms (Control Day 2: n=53, D-Alanine: n=15, β-Alanine: n=14, D-Arginine: n=13). (C-E) Average traces for AVM response in drug-treated animals that do not show a significant difference from the control groups. (C) Day 1 adult worms (Control Day 1: n=53, D-Glutamic acid: n=10, D-Serine: n=12), (D) Day 2 adult worms (Control Day 2: n=53, γ-D-Glutamylglycine: n=10, D-Asparagine: n=4), and (E) Day 3 adult worms (Control Day 3: n=35, 1,1'-Ethylidene-bis(L-tryptophan): n=10, D-Cysteine: n=10, D-Glutamine: n=11, D-Histidine: n=13).

Number	Name	Rationale	Sample size	Number of responding worms
1	D-Glutamic acid	Putative endogenous ligand	10	9
2	D-Serine	Putative endogenous ligand	12	8
3	D-Isoleucine	D-Amino acid	10	7
4	D-Lysine	D-Amino acid	10	8
5	D-Alanine	D-Amino acid	15	7
6	β-Alanine	Endogenous	14	11
7	γ-D-Glutamylglycine	D-Amino acid	10	9
8	D-Arginine	D-Amino acid	13	6
9	D-Asparagine	D-Amino acid	4	4
10	1,1'-Ethylidene-bis(L-tryptophan)	Bioactive tryptophan derivative	10	8
11	D-Cysteine	D-Amino acid	10	6
12	D-Glutamine	D-Amino acid	11	6
13	D-Histidine	D-Amino acid	13	12

Table S1. The 13 compounds of the orphan library were used for the drug screen. Sample size is the total number of tested worms and if the value of maximum responses is larger than 0.5, it is counted as a responding worm.

Video 1: Calcium dynamics of AVM neuron to 0.5s anterior stimulation at 20psi. Stimulus was delivered 10s after recording baseline of neuronal activity. The transgenic animal shown here expresses GCaMP6 and RFP in AVM neuron (*ljIs142[mec-4::GCaMP6m::SL2TagRFP, unc-119] II; unc-119(ed3) III*). Left panel shows green fluorescence from GCaMP6m (left) and red fluorescence from RFP (right) in false colors. White boxes indicate location of AVM neuron and shows how algorithm tracks the neuron. Right graph shows the quantitative calcium trance and red circle indicates the current time point of video. Stimulus occurs at 10s (red dash line). 5x playback.

Video 2: Calcium dynamics of PLM neuron to 1s posterior stimulation at 45psi. Stimulus was delivered 10s after recording baseline of neuronal activity. The transgenic animal shown here expresses GCaMP6 and RFP in PLM neuron (*ljIs142[mec-4::GCaMP6m::SL2TagRFP, unc-119] II; unc-119(ed3) III*). Left panel shows green fluorescence from GCaMP6m (left) and red fluorescence from RFP (right) in false colors. White boxes indicate location of PLM neuron and shows how algorithm tracks the neuron. Right graph shows the quantitative calcium trance and red circle indicates the current time point of video. Stimulus occurs at 10s (red dash line). 5x playback.

Video 3: Calcium dynamics of AVM neuron to 1s anterior stimulation at 45psi. Stimulus was delivered 10s after recording baseline of neuronal activity. The transgenic animal shown here expresses GCaMP6 and RFP in AVM neuron (*ljIs142[mec-4::GCaMP6m::SL2TagRFP, unc-119] II; unc-119(ed3) III*). Left panel shows green fluorescence from GCaMP6m (left) and red fluorescence from RFP (right) in false colors. White boxes indicate location of AVM neuron and shows how algorithm tracks the neuron. Right graph shows the quantitative calcium trance and red circle indicates the current time point of video. Stimulus occurs at 10s (red dash line). 5x playback.

Video 4: Calcium dynamics of PVD neuron to 1s posterior stimulation at 45psi. Stimulus was delivered 10s after recording baseline of neuronal activity. The transgenic animal shown here expresses GCaMP6 in PVD neuron (*wyls5007[ser2prom3::GCaMP6, egl-17::mCherry] X*). Left panel shows green fluorescence from GCaMP6 in false color. A white box indicates location of PVD neuron and shows how algorithm tracks the neuron. Right graph shows the quantitative

calcium trance and red circle indicates the current time point of video. Stimulus occurs at 10s (red dash line). 5x playback.
